# Observer-study-based approaches to quantitatively evaluate the realism of synthetic medical images

**DOI:** 10.1088/1361-6560/acc0ce

**Published:** 2023-03-21

**Authors:** Ziping Liu, Scott Wolfe, Zitong Yu, Richard Laforest, Joyce C Mhlanga, Tyler J Fraum, Malak Itani, Farrokh Dehdashti, Barry A Siegel, Abhinav K Jha

**Affiliations:** 1 Department of Biomedical Engineering, Washington University, St. Louis, MO 63130, United States of America; 2 Mallinckrodt Institute of Radiology, Washington University School of Medicine, St. Louis, MO 63110, United States of America; 3 Alvin J. Siteman Cancer Center, Washington University School of Medicine, St. Louis, MO 63110, United States of America

**Keywords:** image synthesis, image quality assessment, medical imaging, observer study

## Abstract

*Objective.* Synthetic images generated by simulation studies have a well-recognized role in developing and evaluating imaging systems and methods. However, for clinically relevant development and evaluation, the synthetic images must be clinically realistic and, ideally, have the same distribution as that of clinical images. Thus, mechanisms that can quantitatively evaluate this clinical realism and, ideally, the similarity in distributions of the real and synthetic images, are much needed. *Approach.* We investigated two observer-study-based approaches to quantitatively evaluate the clinical realism of synthetic images. In the first approach, we presented a theoretical formalism for the use of an ideal-observer study to quantitatively evaluate the similarity in distributions between the real and synthetic images. This theoretical formalism provides a direct relationship between the area under the receiver operating characteristic curve, AUC, for an ideal observer and the distributions of real and synthetic images. The second approach is based on the use of expert-human-observer studies to quantitatively evaluate the realism of synthetic images. In this approach, we developed a web-based software to conduct two-alternative forced-choice (2-AFC) experiments with expert human observers. The usability of this software was evaluated by conducting a system usability scale (SUS) survey with seven expert human readers and five observer-study designers. Further, we demonstrated the application of this software to evaluate a stochastic and physics-based image-synthesis technique for oncologic positron emission tomography (PET). In this evaluation, the 2-AFC study with our software was performed by six expert human readers, who were highly experienced in reading PET scans, with years of expertise ranging from 7 to 40 years (median: 12 years, average: 20.4 years). *Main results.* In the ideal-observer-study-based approach, we theoretically demonstrated that the AUC for an ideal observer can be expressed, to an excellent approximation, by the Bhattacharyya distance between the distributions of the real and synthetic images. This relationship shows that a decrease in the ideal-observer AUC indicates a decrease in the distance between the two image distributions. Moreover, a lower bound of ideal-observer AUC = 0.5 implies that the distributions of synthetic and real images exactly match. For the expert-human-observer-study-based approach, our software for performing the 2-AFC experiments is available at https://apps.mir.wustl.edu/twoafc. Results from the SUS survey demonstrate that the web application is very user friendly and accessible. As a secondary finding, evaluation of a stochastic and physics-based PET image-synthesis technique using our software showed that expert human readers had limited ability to distinguish the real images from the synthetic images. *Significance.* This work addresses the important need for mechanisms to quantitatively evaluate the clinical realism of synthetic images. The mathematical treatment in this paper shows that quantifying the similarity in the distribution of real and synthetic images is theoretically possible by using an ideal-observer-study-based approach. Our developed software provides a platform for designing and performing 2-AFC experiments with human observers in a highly accessible, efficient, and secure manner. Additionally, our results on the evaluation of the stochastic and physics-based image-synthesis technique motivate the application of this technique to develop and evaluate a wide array of PET imaging methods.

## 1. Introduction

In medical imaging, the use of simulation studies to develop and objectively evaluate new and improved imaging methods has been well recognized (Frangi *et al* 2018, Abadi *et al* 2020, Jha *et al* 2021, 2022, Yousefirizi *et al* 2021). Simulation studies offer the advantage of evaluating the performance of a method against known ground truth, provide the ability to accurately model patient anatomy and physiology as well as imaging system characteristics, incorporate population variability, and generate multiple scan realizations of the same patient to evaluate reproducibility. Even more importantly, this is all done in silico, which is inexpensive and enables optimizing the method before conducting clinical studies. Given these advantages, simulation studies have been used to evaluate a wide range of imaging methods for system instrumentation (Surti *et al* 2006), image reconstruction (Song *et al* 2011), image enhancement (Yu *et al* 2020), and image segmentation (Liu *et al* 2022). Further, the advantages of simulation studies have led to the emergence of virtual clinical trial-based frameworks to evaluate imaging methods (Maidment 2014, Badano *et al* 2018, Abadi *et al* 2020, Badano 2021, Li *et al* 2022). Simulation studies have also shown promise in developing artificial intelligence (AI)-based algorithms for medical imaging. More specifically, a key challenge in developing AI-based algorithms is the requirement of large amounts of training data with known ground truth. This data can be difficult, expensive, and time-consuming to obtain, thus creating a barrier to developing learning-based algorithms. Studies have shown that synthetic images generated from simulations can help alleviate this requirement by providing such training data for purposes such as pre-training the network (Chartsias *et al* 2017a, Creswell *et al* 2018, Gong *et al* 2018, Guan and Loew 2019, Leung *et al* 2020).

For the simulation-based development and evaluation studies to yield clinically relevant inferences, it is important that images generated by the synthesis techniques are clinically realistic (Song *et al* 2011, Jha *et al* 2016, 2021). Ensuring this clinical realism requires that patient anatomy and physiology, population variability, and imaging-system physics are all modeled accurately. There has been much work on evaluating the accuracy in modeling the imaging physics (Gonias *et al* 2007, Poon *et al* 2015, Hernandez-Giron *et al* 2019). However, fewer studies have focused on developing approaches to ensure that the population variability is modeled accurately (Badano *et al* 2018, Zhou *et al* 2019a, Houbrechts *et al* 2021). Note that to ensure clinical realism, it is not sufficient to just assess whether the real and synthetic images match for one patient realization. Instead, for clinically relevant studies, the ideal goal is that the distributions of real and synthetic images should match. This provides confidence that the findings of objective evaluation studies with synthetic images, including virtual clinical trials, are clinically relevant. Further, the clinical realism of synthetic images has been observed to be necessary when using these images for pre-training AI-based algorithms (Leung *et al* 2020). Thus, there is an important need for mechanisms that can quantitatively evaluate the clinical realism of synthetic images and, ideally, the similarity in distributions of real and synthetic images. To address this need, we present two observer-study-based approaches in this manuscript, one based on the ideal observer and the other based on the human observer.

To quantify the distance between distributions of real and synthetic images, metrics such as the Fréchet inception distance (FID) (Heusel *et al* 2017) have been proposed. The FID measures the difference between the statistics extracted from real and synthetic images using a pre-trained Inception network. However, this network is typically pre-trained on ImageNet, which comprises only natural images. Thus, it is unclear whether the network can effectively generalize to evaluate the realism of synthetic medical images. Another set of metrics attempt to evaluate the difference between distributions of real and synthetic images based on the performance of an image classifier (Shmelkov *et al* 2018). These approaches, while promising, rely on the choice of the classifier. More importantly, it is theoretically unclear whether this performance relates to the similarity in distributions between the real and synthetic images.

More recently, observer-study-based approaches have been considered to evaluate the clinical realism of synthetic images (Burgess 2011, Chen *et al* 2016, Elangovan *et al* 2017, Ma *et al* 2017, Sturgeon *et al* 2017). In these approaches, a two-alternative forced-choice (2-AFC) experiment is typically performed. In this 2-AFC experiment, an observer is presented pairs of real and synthetic images. For each image pair, the observer is asked to identify the real image. It is well accepted that the probability of correctly identifying the real image is equivalent to the area under the receiver operating characteristics curve, AUC, for that observer (Barrett and Myers 2013). Thus, if an observer correctly identifies the real images for only 50% of the cases, this yields an AUC of 0.5. Consequently, this implies that the observer is unable to differentiate the real images from the synthetic images. However, this does not necessarily indicate that the distribution of synthetic images matches that of real images. To illustrate this point, we consider a numerical observer. This observer, in the 2-AFC experiment, calculates a test statistic for each image and identifies the image that yields a higher value of test statistic as real. However, the test statistic is just a single statistic derived from the entire image. Thus, while an AUC of 0.5 may indicate that the distributions of the test statistic of the real and synthetic images match, this does not necessarily indicate that distributions of the real and synthetic images also match. Further, when the AUC value is greater than 0.5, it is unclear how the AUC value relates to the distance between the distributions of real and synthetic images. A mathematical analysis for answering these questions is much needed.

The first goal of this work is to theoretically demonstrate that an ideal observer provides a mechanism to quantify the similarity in distributions between the real and synthetic images. This ideal observer, also referred to as the likelihood-ratio test, uses all the statistical information available in the data to maximize task performance. Further, this observer is numerical and, thus, paves the way for a mathematical analysis. In this context, in 1998, Barrett *et al* (1998) published a seminal paper with the goal of bridging the gap between the use of signal-to-noise ratio and the use of the AUC as a figure of merit for signal-detection tasks. In that paper, one of the important findings was deriving the AUC for an ideal observer explicitly in terms of the distributions of signal-present and signal-absent images. By following a similar mathematical treatment as in Barrett *et al*, but in the context of evaluating the clinical realism of synthetic images, we show that an ideal-observer-study-based approach can be used to quantitatively assess the similarity in distributions of the real and synthetic images (section 2). Specifically, we show that the ideal-observer AUC is related, to an excellent approximation, to the Bhattacharyya distance (Bhattacharyya 1943) between the distributions of the real and synthetic images.

The second goal of this work is to develop an openly-available web-based platform to evaluate the clinical realism of synthetic images using human-observer studies. In this context, a vast majority of observer-study-based approaches to evaluate the clinical realism of synthetic images have relied on the use of human observers (Burgess 2011, Chen *et al* 2016, Elangovan *et al* 2017, Ma *et al* 2017, Sturgeon *et al* 2017). Among the different human observers, physicians have multiple years of experience reading medical images and are very familiar with the intricate details of these images. Thus, these physicians, whom we refer to as expert human observers, are best placed to identify even minute differences between the real and synthetic images. To conduct observer studies with expert human readers, various software have been developed. However, these software often require manual installation on local workstations with compatible operating systems (Håkansson *et al* 2010, Zhang *et al* 2016, Genske and Jahnke 2022). The variety in existing operating systems and the fact that users must obtain administrative privileges to install software on workstations owned by institution limit the accessibility of those software. Consequently, these factors make it challenging and cumbersome to conduct human-observer studies. Thus, an accessible and easy-to-use tool that can facilitate the conducting of expert-human-observer studies for evaluating the realism of synthetic images is much needed. Our developed web-based platform (section 3) is in the direction of addressing this need.

## 2. Ideal-observer-study-based approach to quantitatively evaluate the similarity in the distributions of real and synthetic images

### 2.1. Problem formulation

Consider a set of clinical images that are acquired from a population of patients scanned by a medical-imaging system. Denote the image of each patient by an *M*-dimensional vector, ${\hat{{\bf{f}}}}^{r}$, which, we assume, lies within the Hilbert space of Euclidean vectors, denoted by ${{\mathbb{E}}}^{M}$. Additionally, consider an image-synthesis method that generates images of a simulated population of patients in silico. Each synthetic medical image, denoted by an *M*-dimensional vector, ${\hat{{\bf{f}}}}^{s}$, is also assumed to lie within ${{\mathbb{E}}}^{M}$.

To evaluate the clinical realism of those synthetic images, we consider a 2-AFC experiment being performed by a numerical observer. In this experiment, an observer is presented with pairs of real and synthetic images, ${\hat{{\bf{f}}}}^{r}$ and ${\hat{{\bf{f}}}}^{s}$. The classes of synthetic and real images are referred to as the hypotheses *H*_1_ and *H*_2_, respectively. Denote the probability of observing an image $\hat{{\bf{f}}}$ under the hypothesis *H*_*j*_ by $\mathrm{pr}(\hat{{\bf{f}}}| {H}_{j})$. Then, ${\hat{{\bf{f}}}}^{s}$ is sampled from $\mathrm{pr}(\hat{{\bf{f}}}| {H}_{1})$ and ${\hat{{\bf{f}}}}^{r}$ is sampled from $\mathrm{pr}(\hat{{\bf{f}}}| {H}_{2})$. The observer is then required to identify the real image. To make this decision, the observer calculates two test statistics, $\theta ({\hat{{\bf{f}}}}^{s})$ and $\theta ({\hat{{\bf{f}}}}^{r})$, and assigns the image that yields the higher value of the test statistic to *H*_2_. The decision is correct if $\theta ({\hat{{\bf{f}}}}^{r})\gt \theta ({\hat{{\bf{f}}}}^{s})$. For convenience of notation, let ${q}_{j}(\hat{{\bf{f}}})\equiv \mathrm{pr}(\hat{{\bf{f}}}| {H}_{j})$. The probability of a correct decision can be calculated as

\begin{eqnarray*}\Pr \left[\theta ({\hat{{\bf{f}}}}^{r})\gt \theta ({\hat{{\bf{f}}}}^{s})\right]={\int }_{\infty }{{\mathrm{d}}}^{M}{\hat{{\bf{f}}}}^{s}{\int }_{\infty }{{\mathrm{d}}}^{M}{\hat{{\bf{f}}}}^{r}\ {q}_{1}({\hat{{\bf{f}}}}^{s}){q}_{2}({\hat{{\bf{f}}}}^{r})\ \mathrm{step}\left(\theta ({\hat{{\bf{f}}}}^{r})-\theta ({\hat{{\bf{f}}}}^{s})\right),\end{eqnarray*}

where $\mathrm{step}\left(\cdot \right)$ denotes the Heaviside unit step function. As shown in Barrett and Myers (2013) in the context of signal-detection tasks and rephrased in this scenario of using the 2-AFC experiment to evaluate the clinical realism of synthetic images (appendix A), the right-hand side of the above expression is equivalent to the expression for the AUC for an observer in terms of integrals over ${\hat{{\bf{f}}}}^{r}$ and ${\hat{{\bf{f}}}}^{s}$. Thus, from equation (1), the accuracy of an observer in identifying the real images in a 2-AFC experiment is equivalent to the AUC for that observer.

We note that the expression for the AUC using equation (1) depends on the test statistics and, thus, does not specify a direct relationship between the AUC value and the distance between the distributions of the real and synthetic images. To gain insights into this relationship, we consider the use of an ideal observer, which uses all the statistical information available in the data to evaluate the realism of synthetic images. This ideal observer sets an upper bound on the performance of any available observers and provides the best ability to assess whether any differences exist between the distributions of the real and synthetic images.

An ideal observer is defined as a decision strategy that calculates the likelihood ratio of ${q}_{2}(\hat{{\bf{f}}})$ and ${q}_{1}(\hat{{\bf{f}}})$ and compares the ratio to a threshold. In other words, the ideal observer calculates the test statistic, Λ, given by

\begin{eqnarray*}{\mathrm{\Lambda }}=\displaystyle \frac{{q}_{2}(\hat{{\bf{f}}})}{{q}_{1}(\hat{{\bf{f}}})}.\end{eqnarray*}

Our goal is to relate the AUC for this ideal observer to the distance between the distributions of ${q}_{1}(\hat{{\bf{f}}})$ and ${q}_{2}(\hat{{\bf{f}}})$.

Toward this goal, a central component of our derivation is the use of a likelihood-generating function (Barrett *et al* 1998). We first provide the background for the likelihood-generating function in section 2.2. We show that the characteristic functions, which are used to obtain the ideal-observer AUC, can be derived solely based on the likelihood-generating function. Then, in section 2.3, we show that the ideal-observer AUC can be expressed, to an excellent approximation, by the likelihood-generating function evaluated at the origin. More importantly, this value at the origin relates directly to the Bhattacharyya distance between the distributions of the real and the synthetic images. Thus, by using the likelihood-generating function, we are able to establish a direct relationship between the ideal-observer AUC and the similarity in distributions of the real and the synthetic images.

### 2.2. Background for likelihood-generating function

The likelihood-generating function is central to our derivation as all moments of both Λ and its logarithm, denoted by *λ*, under hypotheses *H*_1_ and *H*_2_ can be derived. This function was originally introduced by Barrett *et al* (1998), and we follow a similar approach to define the function. Denote the expectation of a random variable *t* under hypothesis *H*_*j*_ by 〈*t*〉_*j*_. We can show that the moments of Λ under *H*_2_ are related to those under *H*_1_ by

\begin{eqnarray*}\langle {{\mathrm{\Lambda }}}^{k}{\rangle }_{2}={\int }_{\infty }{{\mathrm{d}}}^{M}\hat{{\bf{f}}}\ {q}_{2}(\hat{{\bf{f}}}){\left[\displaystyle \frac{{q}_{2}(\hat{{\bf{f}}})}{{q}_{1}(\hat{{\bf{f}}})}\right]}^{k}={\int }_{\infty }{{\mathrm{d}}}^{M}\hat{{\bf{f}}}\ {q}_{1}(\hat{{\bf{f}}}){\left[\displaystyle \frac{{q}_{2}(\hat{{\bf{f}}})}{{q}_{1}(\hat{{\bf{f}}})}\right]}^{k+1}=\langle {{\mathrm{\Lambda }}}^{k+1}{\rangle }_{1}.\end{eqnarray*}

Since ${\mathrm{\Lambda }}=\exp (\lambda )$, we can re-write equation (3) as

\begin{eqnarray*}\langle \exp \left(k\lambda \right){\rangle }_{2}=\langle \exp \left[(k+1)\lambda \right]{\rangle }_{1}.\end{eqnarray*}

The moment-generating function for a random variable *t* under hypothesis *H*_*j*_, denoted by *M*_*j*_(*β*), is defined by

\begin{eqnarray*}{M}_{j}(\beta )={\int }_{-\infty }^{\infty }{\mathrm{d}}{t}\ \mathrm{pr}(t| {H}_{j})\exp (\beta t)=\langle \exp (\beta t){\rangle }_{j}.\end{eqnarray*}

Thus, from equation (4), the relationship between the moment-generating functions under the two hypotheses is given by:

\begin{eqnarray*}{M}_{2}(\beta )={M}_{1}(\beta +1).\end{eqnarray*}

Additionally, the characteristic function for a random variable *t* under hypothesis *H*_*j*_, denoted by *ψ*_*j*_(*ξ*), is defined by

\begin{eqnarray*}{\psi }_{j}(\xi )={\int }_{-\infty }^{\infty }{\mathrm{d}}{t}\ \mathrm{pr}(t| {H}_{j})\exp \left(-2\pi {\mathrm{i}}\xi t\right).\end{eqnarray*}

From equations (5) and (7), we readily see that the moment-generating functions and characteristic functions are related to each other by

\begin{eqnarray*}{M}_{j}(\beta )={\psi }_{j}\left(\displaystyle \frac{{\mathrm{i}}\beta }{2\pi }\right).\end{eqnarray*}

Then, using equations (6) and (8) yields the relationship between the characteristic functions for *λ* under hypotheses *H*_1_ (class of synthetic images) and *H*_2_ (class of real images):

\begin{eqnarray*}{\psi }_{2}(\xi )={\psi }_{1}\left(\xi +\displaystyle \frac{{\mathrm{i}}}{2\pi }\right).\end{eqnarray*}

This equation is important since it can further be used to derive the relationship between the probability distributions of *λ* under the two hypothesis. Denote the probability distribution of *λ* under hypothesis *H*_*j*_ by *p*_*j*_(*λ*). Applying inverse Fourier transform to equation (9) on both sides yields (appendix B)

\begin{eqnarray*}{p}_{2}(\lambda )=\exp \left(\lambda \right){p}_{1}(\lambda ).\end{eqnarray*}

In equation (10), both *p*_1_(*λ*) and *p*_2_(*λ*) can be derived from a single non-negative function *f*(*λ*), as follows:

\begin{eqnarray*}{p}_{1}(\lambda )=\exp \left(-\displaystyle \frac{1}{2}\lambda \right)f(\lambda ),\end{eqnarray*}

\begin{eqnarray*}{p}_{2}(\lambda )=\exp \left(\displaystyle \frac{1}{2}\lambda \right)f(\lambda ).\end{eqnarray*}

Defining this function *f*(*λ*) can help us to derive the expressions for the moment-generating functions and characteristic functions now. Denote the two-sided Laplace transform of *f*(*λ*) by ${{ \mathcal F }}_{L}(\beta )$, such that

\begin{eqnarray*}{{ \mathcal F }}_{L}(\beta )={\int }_{-\infty }^{\infty }{\mathrm{d}}\lambda \ \exp (\beta \lambda )f(\lambda ).\end{eqnarray*}

Then, from equation (6), we obtain

\begin{eqnarray*}{M}_{1}(\beta )={{ \mathcal F }}_{L}\left(\beta -\displaystyle \frac{1}{2}\right),\end{eqnarray*}

\begin{eqnarray*}{M}_{2}(\beta )={{ \mathcal F }}_{L}\left(\beta +\displaystyle \frac{1}{2}\right).\end{eqnarray*}

Similarly, *ψ*_1_(*ξ*) and *ψ*_2_(*ξ*) in equation (9) can be expressed in terms of the Fourier transform of *f*(*λ*), denoted by ${ \mathcal F }(\xi )$:

\begin{eqnarray*}{\psi }_{1}(\xi )={ \mathcal F }\left(\xi -\displaystyle \frac{{\mathrm{i}}}{4\pi }\right),\end{eqnarray*}

\begin{eqnarray*}{\psi }_{2}(\xi )={ \mathcal F }\left(\xi +\displaystyle \frac{{\mathrm{i}}}{4\pi }\right).\end{eqnarray*}

The term *p*_*j*_(*λ*) denotes a probability and should integrate to unity. Thus, from equations (13) and (14), ${{ \mathcal F }}_{L}\left(\beta \pm \tfrac{1}{2}\right)$ and ${ \mathcal F }\left(\xi \pm \tfrac{{\mathrm{i}}}{4\pi }\right)$ should equal to unity. To enforce these constraints, the likelihood-generating function *G*(*β*) and another function *T*(*ξ*) are defined such that

\begin{eqnarray*}{{ \mathcal F }}_{L}(\beta )=\exp \left[\left(\beta +\displaystyle \frac{1}{2}\right)\left(\beta -\displaystyle \frac{1}{2}\right)G(\beta )\right],\end{eqnarray*}

\begin{eqnarray*}{ \mathcal F }(\xi )=\exp \left[\left(\xi +\displaystyle \frac{{\mathrm{i}}}{4\pi }\right)\left(\xi -\displaystyle \frac{{\mathrm{i}}}{4\pi }\right)T(\xi )\right].\end{eqnarray*}

We can then express *M*_1_(*β*) and *ψ*_1_(*ξ*) as

\begin{eqnarray*}{M}_{1}(\beta )=\exp \left[\beta (\beta -1)G(\beta -\displaystyle \frac{1}{2})\right],\end{eqnarray*}

\begin{eqnarray*}{\psi }_{1}(\xi )=\exp \left[\xi (\xi -\displaystyle \frac{{\mathrm{i}}}{2\pi })T(\xi -\displaystyle \frac{{\mathrm{i}}}{4\pi })\right].\end{eqnarray*}

Additionally, from equation (8), *T*(*ξ*) can be expressed in terms of *G*(*β*):

\begin{eqnarray*}T(\xi )=-4{\pi }^{2}G(-2\pi {\mathrm{i}}\xi ).\end{eqnarray*}

Thus, we see that the characteristic functions can be expressed using only the likelihood-generating function.

### 2.3. Deriving the relationship between the ideal-observer AUC and the similarity in distributions of the real and the synthetic images

Having obtained the characteristic functions using the likelihood-generating function, we can now derive the expression for the ideal-observer AUC. For this purpose, we note from equation (1) that by expressing the step function in terms of its Fourier transform, we can calculate the AUC as

\begin{eqnarray*}\mathrm{AUC}=\displaystyle \frac{1}{2}+\displaystyle \frac{1}{2\pi {\mathrm{i}}}{\mathscr{P}}{\int }_{-\infty }^{\infty }\displaystyle \frac{{\mathrm{d}}\xi }{\xi }{\int }_{\infty }{{\mathrm{d}}}^{M}{\hat{{\bf{f}}}}^{s}{\int }_{\infty }{{\mathrm{d}}}^{M}{\hat{{\bf{f}}}}^{r}\ {q}_{1}({\hat{{\bf{f}}}}^{s}){q}_{2}({\hat{{\bf{f}}}}^{r})\exp \left\{2\pi {\mathrm{i}}\xi \left[\theta ({\hat{{\bf{f}}}}^{r})-\theta ({\hat{{\bf{f}}}}^{s})\right]\right\}\end{eqnarray*}

\begin{eqnarray*}\begin{array}{rcl} &amp; = &amp; \displaystyle \frac{1}{2}+\displaystyle \frac{1}{2\pi {\mathrm{i}}}{\mathscr{P}}{\int }_{-\infty }^{\infty }\displaystyle \frac{{\mathrm{d}}\xi }{\xi }\left\{{\int }_{\infty }{{\mathrm{d}}}^{M}{\hat{{\bf{f}}}}^{s}\ {q}_{1}({\hat{{\bf{f}}}}^{s})\exp \left[-2\pi {\mathrm{i}}\xi \theta ({\hat{{\bf{f}}}}^{s})\right]\right\}\\ &amp; &amp; \times \left\{{\int }_{\infty }{{\mathrm{d}}}^{M}{\hat{{\bf{f}}}}^{r}\ {q}_{2}({\hat{{\bf{f}}}}^{r})\exp \left[2\pi {\mathrm{i}}\xi \theta ({\hat{{\bf{f}}}}^{r})\right]\right\},\end{array}\end{eqnarray*}

where ${\mathscr{P}}$ denotes the Cauchy principal value for evaluating the improper integral. Note that in equation (18*b*), the expression within each curly bracket is the same as calculating the expectation of the term $(\pm )2\pi {\mathrm{i}}\xi \theta (\hat{{\bf{f}}})$. Using the fact that this expectation can be calculated from the probability density on either $\hat{{\bf{f}}}$ or $\theta (\hat{{\bf{f}}})$, we can further write equation (18*b*) in terms of the characteristic functions (equation (7)) as

\begin{eqnarray*}\mathrm{AUC}=\displaystyle \frac{1}{2}+\displaystyle \frac{1}{2\pi {\mathrm{i}}}{\mathscr{P}}{\int }_{-\infty }^{\infty }\displaystyle \frac{{\mathrm{d}}\xi }{\xi }\ {\psi }_{1}(\xi ){\psi }_{2}^{* }(\xi ).\end{eqnarray*}

By replacing the expression for *ψ*_2_(*ξ*) from equation (9) and using the Hermiticity property of the Fourier transform, we obtain

\begin{eqnarray*}\mathrm{AUC}=\displaystyle \frac{1}{2}+\displaystyle \frac{1}{2\pi {\mathrm{i}}}{\mathscr{P}}{\int }_{-\infty }^{\infty }\displaystyle \frac{{\mathrm{d}}\xi }{\xi }{\psi }_{1}(\xi ){\psi }_{1}\left(-\xi +\displaystyle \frac{{\mathrm{i}}}{2\pi }\right)\end{eqnarray*}

\begin{eqnarray*}=\,\,\displaystyle \frac{1}{2}+\displaystyle \frac{1}{2\pi {\mathrm{i}}}{\mathscr{P}}{\int }_{-\infty }^{\infty }\displaystyle \frac{{\mathrm{d}}\xi }{\xi }\exp \left\{-4{\pi }^{2}\left({\xi }^{2}-\displaystyle \frac{{\mathrm{i}}\xi }{2\pi }\right)\left[G(2\pi {\mathrm{i}}\xi +\displaystyle \frac{1}{2})+G\left(-2\pi {\mathrm{i}}\xi -\displaystyle \frac{1}{2}\right)\right]\right\},\end{eqnarray*}

where, in the second step, we have used the expression for *ψ*_1_(*ξ*) from equation (16*b*) and then the relationship between *T*(*ξ*) and *G*(*β*) from equation (17). To simplify this further, we can approximate *G*(*β*) via the Maclaurin series expansion:

\begin{eqnarray*}G(\beta )=\displaystyle \sum _{n=0}^{\infty }{G}^{(n)}(0)\displaystyle \frac{{\beta }^{n}}{n!}.\end{eqnarray*}

Substituting this in equation (20*b*) and assuming that the contribution of higher order (*n* > 1) terms is negligible yields

\begin{eqnarray*}\mathrm{AUC}=\displaystyle \frac{1}{2}+\displaystyle \frac{1}{2\pi {\mathrm{i}}}{\mathscr{P}}{\int }_{-\infty }^{\infty }\displaystyle \frac{{\mathrm{d}}\xi }{\xi }\exp \left\{-4{\pi }^{2}\left({\xi }^{2}-\displaystyle \frac{{\mathrm{i}}\xi }{2\pi }\right)\displaystyle \sum _{n=0}^{\infty }{G}^{(n)}(0)\displaystyle \frac{{\left(2\pi {\mathrm{i}}\xi +\tfrac{1}{2}\right)}^{n}+{\left(-2\pi {\mathrm{i}}\xi -\tfrac{1}{2}\right)}^{n}}{n!}\right\}\end{eqnarray*}

\begin{eqnarray*}=\,\,\displaystyle \frac{1}{2}+\displaystyle \frac{1}{2\pi {\mathrm{i}}}{\mathscr{P}}{\int }_{-\infty }^{\infty }\displaystyle \frac{{\mathrm{d}}\xi }{\xi }\exp \left\{-4{\pi }^{2}\left({\xi }^{2}-\displaystyle \frac{{\mathrm{i}}\xi }{2\pi }\right)\displaystyle \sum _{k=0}^{\infty }2{G}^{(2k)}(0)\displaystyle \frac{{\left(2\pi {\mathrm{i}}\xi +\tfrac{1}{2}\right)}^{2k}}{(2k)!}\right\}\end{eqnarray*}

\begin{eqnarray*}\approx \,\displaystyle \frac{1}{2}+\displaystyle \frac{1}{2\pi {\mathrm{i}}}{\mathscr{P}}{\int }_{-\infty }^{\infty }\displaystyle \frac{{\mathrm{d}}\xi }{\xi }\exp \left\{-4{\pi }^{2}\left({\xi }^{2}-\displaystyle \frac{{\mathrm{i}}\xi }{2\pi }\right)\times 2G(0)\right\}.\end{eqnarray*}

By means of tabular integral, equation (22*c*) yields

\begin{eqnarray*}\mathrm{AUC}\approx \,\displaystyle \frac{1}{2}+\displaystyle \frac{1}{2}\mathrm{erf}\left[\displaystyle \frac{1}{2}\sqrt{2G(0)}\right].\end{eqnarray*}

Next, using equations (15*a*), (12), and (11*a*), we obtain

\begin{eqnarray*}G(0)=-4\mathrm{log}{{ \mathcal F }}_{L}(0)\end{eqnarray*}

\begin{eqnarray*}=\,\,-4\mathrm{log}{\int }_{-\infty }^{\infty }{\mathrm{d}}\lambda \ {p}_{1}(\lambda )\exp \left(\displaystyle \frac{1}{2}\lambda \right)\end{eqnarray*}

\begin{eqnarray*}=\,\,-4\mathrm{log}\langle {{\mathrm{\Lambda }}}^{\tfrac{1}{2}}{\rangle }_{1}\end{eqnarray*}

\begin{eqnarray*}=\,\,-4\mathrm{log}\left[{\int }_{\infty }{{\mathrm{d}}}^{M}\hat{{\bf{f}}}\sqrt{{q}_{1}(\hat{{\bf{f}}}){q}_{2}(\hat{{\bf{f}}})}\right]\end{eqnarray*}

\begin{eqnarray*}=\,\,4{D}_{B}({q}_{1}(\hat{{\bf{f}}}),{q}_{2}(\hat{{\bf{f}}})),\end{eqnarray*}

where, in equation (24*e*), the term ${D}_{B}({q}_{1}(\hat{{\bf{f}}}),{q}_{2}(\hat{{\bf{f}}}))$ is the well-known Bhattacharyya distance (Bhattacharyya 1943) that measures the similarity between the distributions ${q}_{1}(\hat{{\bf{f}}})$ and ${q}_{2}(\hat{{\bf{f}}})$. The term ${\int }_{\infty }{{\mathrm{d}}}^{M}\hat{{\bf{f}}}\sqrt{{q}_{1}(\hat{{\bf{f}}}){q}_{2}(\hat{{\bf{f}}})}$ in equation (24*d*) is the Bhattacharyya coefficient. Then, from equations (23) and (24*e*), we obtain that for an ideal observer, the AUC can be approximated excellently in terms of the Bhattacharyya distance between ${q}_{1}(\hat{{\bf{f}}})$ and ${q}_{2}(\hat{{\bf{f}}})$:

\begin{eqnarray*}\mathrm{AUC}\approx \displaystyle \frac{1}{2}+\displaystyle \frac{1}{2}\mathrm{erf}\left[\sqrt{2{D}_{B}({q}_{1}(\hat{{\bf{f}}}),{q}_{2}(\hat{{\bf{f}}}))}\right].\end{eqnarray*}

Note that equation (25) is obtained without making any assumption of the probability law of either the images $\hat{{\bf{f}}}$ or the likelihood ratio Λ.

From equation (25), it is easy to show that the value of the ideal-observer AUC decreases as the Bhattacharyya distance between ${q}_{1}(\hat{{\bf{f}}})$ and ${q}_{2}(\hat{{\bf{f}}})$ decreases, and vice versa. Further, a lower bound of AUC = 0.5 is obtained when the Bhattacharyya distance is at the minimum value of 0, i.e. ${q}_{1}(\hat{{\bf{f}}})$ exactly matches ${q}_{2}(\hat{{\bf{f}}})$. Thus, an ideal-observer-study-based approach provides a mechanism to quantitatively evaluate the similarity in distributions of the real and the synthetic images.

### 2.4. Illustrating the relationship between the ideal-observer AUC and the Bhattacharyya distance for a two-pixel image setup

**Figure 1.**
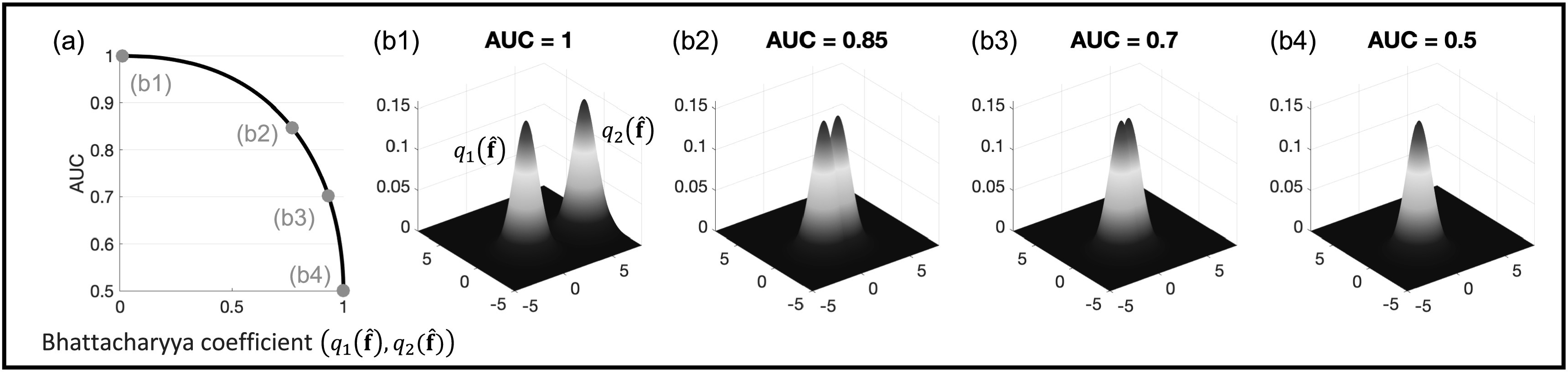
Illustrating the relationship in equation (25) between the ideal-observer AUC and similarity in distributions of ${q}_{1}(\hat{{\bf{f}}})$ and ${q}_{2}(\hat{{\bf{f}}})$ for a two-pixel image setup. (a) The computed AUC values as a function of the Bhattacharyya coefficient between ${q}_{1}(\hat{{\bf{f}}})$ and ${q}_{2}(\hat{{\bf{f}}})$ (equation (24*d*)). (b1-4) The computed AUC values for four representative cases. We note in (b4) that for perfect overlap between ${q}_{1}(\hat{{\bf{f}}})$ and ${q}_{2}(\hat{{\bf{f}}})$, the ideal-observer AUC achieves the lower bound of 0.5.

To illustrate the relationship in equation (25), consider that $\hat{{\bf{f}}}$ denotes images consisting of only two pixels. For the sake of simplicity, assume that ${q}_{1}(\hat{{\bf{f}}})$ and ${q}_{2}(\hat{{\bf{f}}})$ are described by 2D Gaussian distributions that have the same covariance matrix but different means, i.e. ${q}_{1}(\hat{{\bf{f}}})\sim { \mathcal N }({{\boldsymbol{\mu }}}_{1},{\mathrm{\Sigma }})$ and ${q}_{2}(\hat{{\bf{f}}})\sim { \mathcal N }({{\boldsymbol{\mu }}}_{2},{\mathrm{\Sigma }})$. We readily see that the Bhattacharyya distance between ${q}_{1}(\hat{{\bf{f}}})$ and ${q}_{2}(\hat{{\bf{f}}})$ decreases as the difference between ***μ***_1_ and ***μ***_2_ decreases. Using equation (25), we can obtain the AUC at different values of ${D}_{B}({q}_{1}(\hat{{\bf{f}}}),{q}_{2}(\hat{{\bf{f}}}))$. As shown in figure 1, the value of AUC decreases and achieves the lower bound of 0.5 as the overlap between ${q}_{1}(\hat{{\bf{f}}})$ and ${q}_{2}(\hat{{\bf{f}}})$ increases, i.e. ${D}_{B}({q}_{1}(\hat{{\bf{f}}}),{q}_{2}(\hat{{\bf{f}}}))$ approaches 0.

## 3. A web-based expert-human-observer-study-based approach to quantitatively evaluate the clinical realism of synthetic images

As introduced in section 1, human-observer studies have been widely used to evaluate the clinical realism of synthetic images. Among the different human observers, expert human readers, such as physicians who are highly experienced in reading medical images, can identify minute differences between the real and synthetic images. A 2-AFC experiment provides a mechanism to quantify the performance of the expert human observers on this task. If an expert human observer correctly identifies the real images for only around 50% of the cases in the 2-AFC experiment, then, as mentioned in section 2.1 with the proof provided in appendix A, this would indicate an AUC of ∼0.5 on the task of detecting the real image. This would imply that the expert human observer was unable to distinguish between the real and synthetic images, thus, suggesting that the synthetic images are clinically realistic as evaluated by that observer.

While several tools have been developed for conducting human-observer studies (Håkansson *et al* 2010, Zhang *et al* 2016), users often need to manually install the tools on local workstations with compatible operating systems and/or have programming knowledge. These requirements can reduce the accessibility of the tools and consequently, serve as a hurdle in designing and conducting the observer studies. To address these issues, we develop an openly available software for conducting the 2-AFC experiments by expert human observers to quantitatively evaluate the clinical realism of synthetic images. This software is designed to be accessible, secure, and have mechanisms for both designing new 2-AFC experiments by investigators and performing the experiments by expert human observers. To achieve these goals, we design this software to be web-based and with a dual-user ‘Investigator-Reader’ interface. The ‘Investigator interface’ allows an investigator to design a 2-AFC experiment and upload the real and the synthetic images. The ‘Reader interface’ allows the expert human observers recruited by this investigator to perform the 2-AFC experiment. The programming environment for building the software is detailed in appendix C. In the following, we focus on describing the main functionalities of this software and the procedures for the investigator and reader to design and perform the 2-AFC experiment.

### 3.1. Developed software

#### 3.1.1. Investigator interface

**Figure 2.**
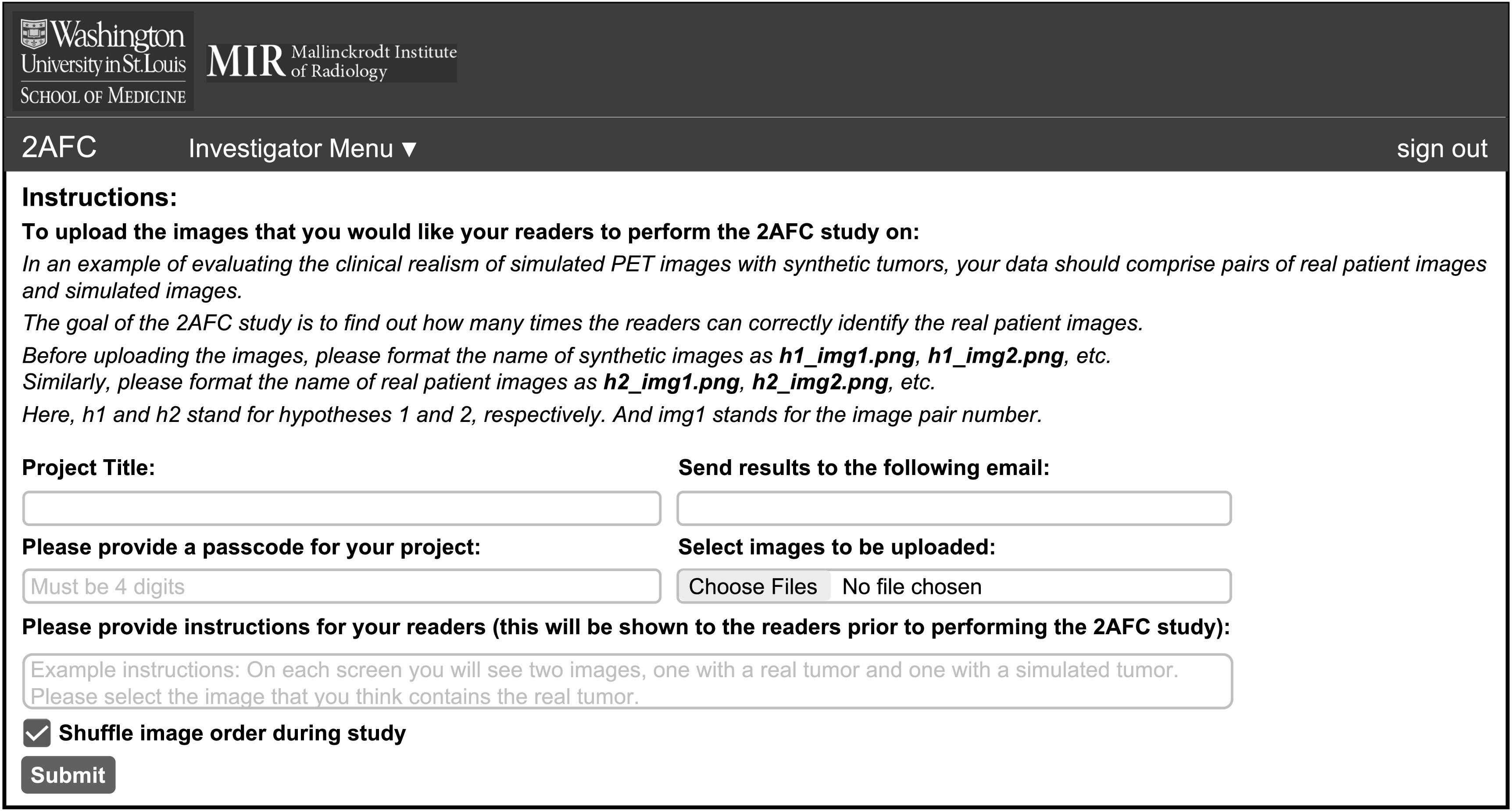
The investigator interface.

The layout for the investigator interface is shown in figure 2. As a first step, the investigator is required to provide a project title and a corresponding four-digit passcode, which the investigator should then share with the readers. This ensures that only readers authorized by this investigator can access the images, thus ensuring the security of the images. To improve the accessibility for readers, the investigator is asked to provide instructions for the readers to perform the 2-AFC experiment on the uploaded images. These instructions will be displayed on the screen once a reader begins the experiment. Our software allows the investigator to upload an arbitrary number of image pairs. The investigator is also provided an option to shuffle the order of image pairs. Finally, the investigator is asked to provide an email address, to which the results of the observer study from each reader would be sent. Note that if an investigator receives results with a percent accuracy much lower than 50%, this is likely an indication that the observer is not trained and, thus, the results should be treated with caution.

#### 3.1.2. Reader interface

**Figure 3.**
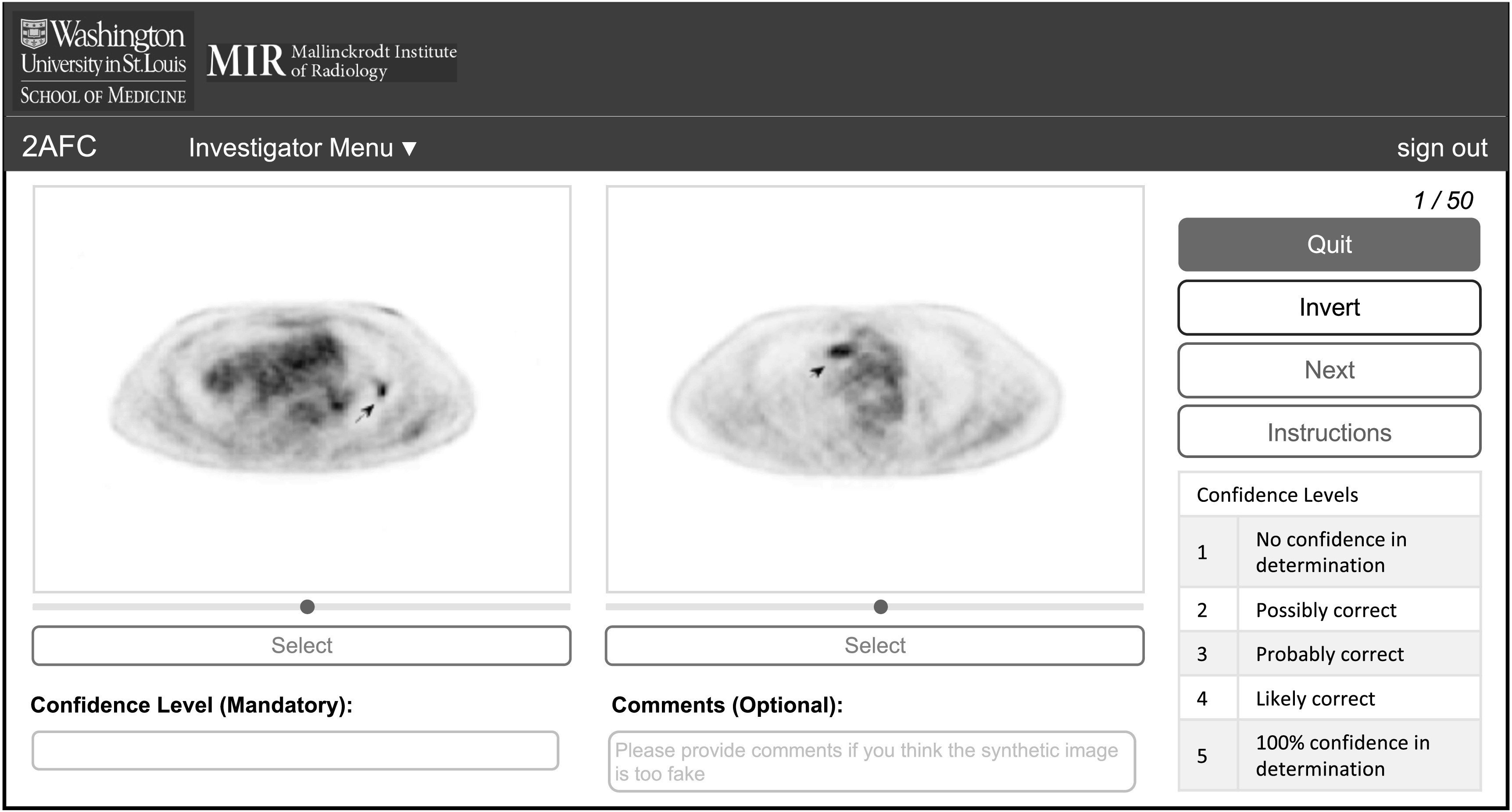
The reader interface.

The reader is required to provide the project title and the corresponding passcode to access the images uploaded by a specific investigator. If these entries are provided correctly, the reader will be directed to the webpage, as shown in figure 3, to perform the 2-AFC experiment. In this experiment, a synthetic image sampled from ${q}_{1}(\hat{{\bf{f}}})$ and a real image sampled from ${q}_{2}(\hat{{\bf{f}}})$ are presented side-by-side (section 2.1). For each image pair, the reader is asked to identify the image that they perceive as real. While making the decision, the reader can adjust the contrast and invert the intensities of the images. The goal of providing these functionalities is to increase the clinical relevance and rigor of the observer study. The reader is also asked to provide a confidence level for the decision. The interpretations of the confidence levels are provided to the reader (figure 3). These interpretations are similar to those used in previous studies to conduct human-observer studies (Chen *et al* 2016, Ma *et al* 2017). The confidence levels could be a useful tool for improving the design of the synthesis technique after the observer-study evaluation. For example, if an expert reader correctly distinguishes the real image from the synthetic image with high confidence level, this could indicate that the synthetic image is highly unrealistic. Investigators could then incorporate such feedback while improving the design of their synthetic-image-generation approaches. Additionally, the reader is provided with an option to leave additional comments.

### 3.2. Evaluating usability of the developed software

To evaluate the usability of our software, we conduct a system usability scale (SUS) survey (Brooke 1996). This survey is widely used to test the usability of newly developed software and websites. The SUS evaluates a software on three main aspects, namely, effectiveness, efficiency, and satisfaction. These aspects assess whether users achieve their goals successfully, the effort and/or resource spent to achieve the goals, and whether the user experience is satisfactory, respectively.

The SUS survey was designed by adapting from Brooke (1996) and consisted of a 10-item questionnaire about the software with five response options for respondents: strongly disagree, disagree, neutral, agree, and strongly agree (table 1). For the odd-numbered items, a score of 0 was assigned to ‘strongly disagree’ and a score of 4 was assigned to ‘strongly agree’. For the even-numbered items, a score of 4 was assigned to ‘strongly disagree’ and a score of 0 was assigned to ‘strongly agree’. The scores were then added, and the summed score was multiplied by 2.5 such that the eventual score fell between 0 and 100.

**Table 1.** The system usability scale (SUS) survey.

Index	Statement
1	I think that I would like to use this software frequently.
2	I found the software unnecessarily complex.
3	I thought the software was easy to use.
4	I think that I would need the support of a technical person to be able to use this software.
5	I found the various functionalities of this software were well integrated.
6	I thought there was too much inconsistency in this software.
7	I would imagine that most people would learn to use this software very quickly.
8	I found the software very cumbersome to use.
9	I felt very confident using the software.
10	I needed to learn a lot of things before I could get going with this software.

We first conducted the survey with five board-certified nuclear medicine physicians with years of expertise ranging from 7 to 40 years (median: 12 years, average: 20.4 years), one nuclear medicine physicist, and one nuclear medicine resident. These users are considered as the expert human observers who would use our software to evaluate the clinical realism of synthetic images. Additionally, we conducted the survey with five users who were asked to evaluate the software as investigators designing an observer study. Conducting the survey with all these users provides evidence for the utility of the software in practical settings.

### 3.3. Evaluating the clinical realism of a positron emission tomography (PET) image-synthesis technique using the developed software

To demonstrate the application of our software to quantitatively evaluate the clinical realism of image-synthesis techniques, we used the software to evaluate a recently developed technique for oncologic PET. This technique is a stochastic and physics-based method that generates 2D^18^ F-fluorodeoxyglucose (FDG)-PET images of patients with lung cancer (Liu *et al* 2021a). By following the simulation procedure detailed in Liu *et al* (2021a), we generated 50 synthetic PET images for our 2-AFC study. The source code for this technique is openly available at https://github.com/ziping-liu/A-stochastic-and-physics-based-method-to-generate-oncological-PET-images.git. Our evaluation study was retrospective, involved clinical images, and was IRB-approved and HIPAA-compliant with informed consent being waived.

The 2-AFC study using our developed software was conducted by six expert readers, including five board-certified PET physicians (BAS, FD, JCM, TJF, and MI) and one PET physicist (RL). The readers were highly experienced in reading PET scans, with years of expertise ranging from 7 to 40 years (median: 16 years, average: 20.3 years). During the study, each of the 50 synthetic images was paired with an existing clinical PET image to be displayed to the readers simultaneously with our software (section 3.1.2; figure 3). The readers were then asked to identify the real image, provide a confidence level for the decision, and optionally leave a comment. We then computed the percentage of times that each reader correctly identified the real PET image.

## 4. Results

### 4.1. Evaluating usability of the developed software for conducting 2-AFC experiments with expert human observers

**Figure 4.**
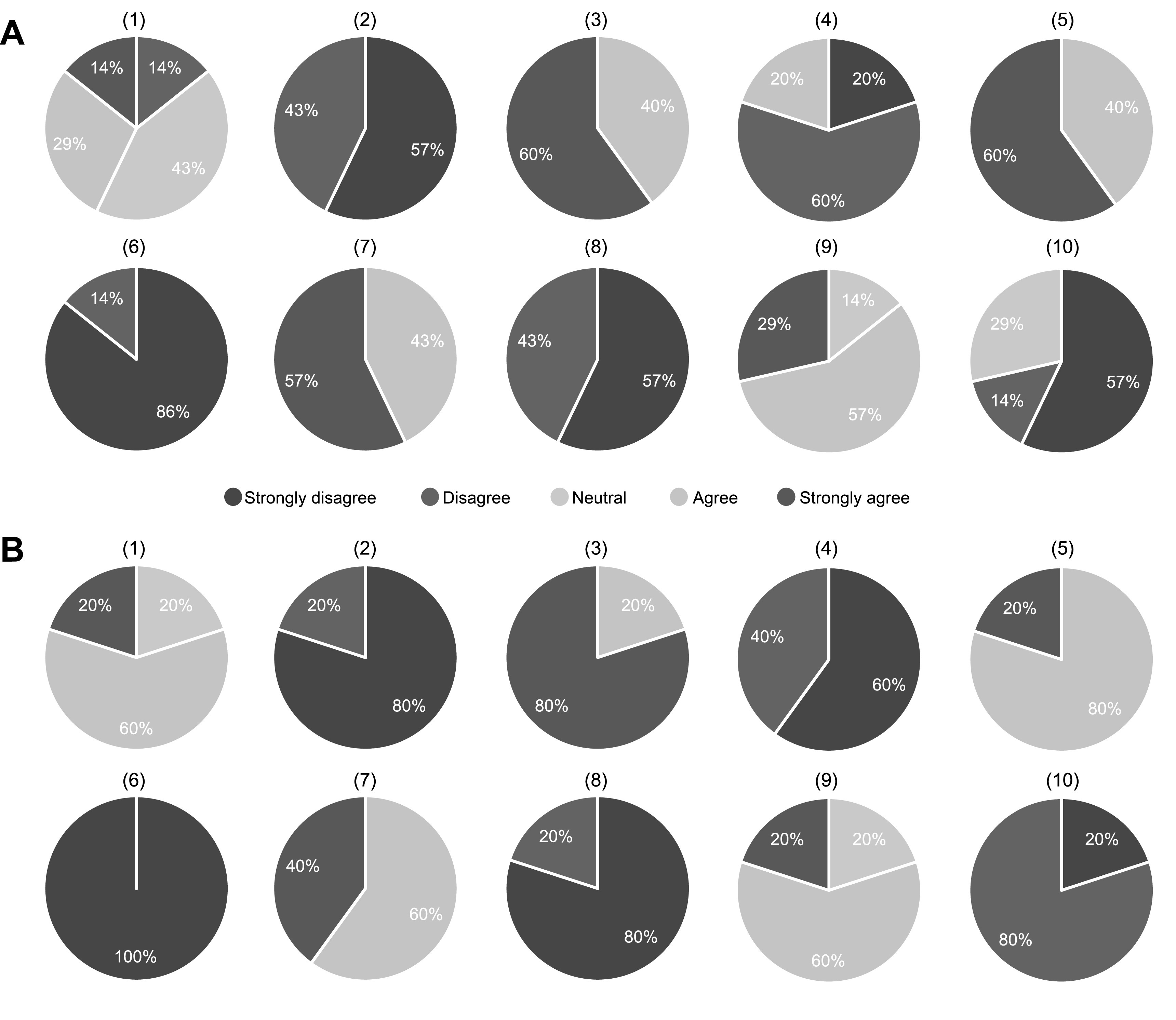
Distribution of responses to each item in the questionnaire from (A) seven expert human readers and (B) five observer-study designers participating in the system usability scale (SUS) survey.

**Figure 5.**
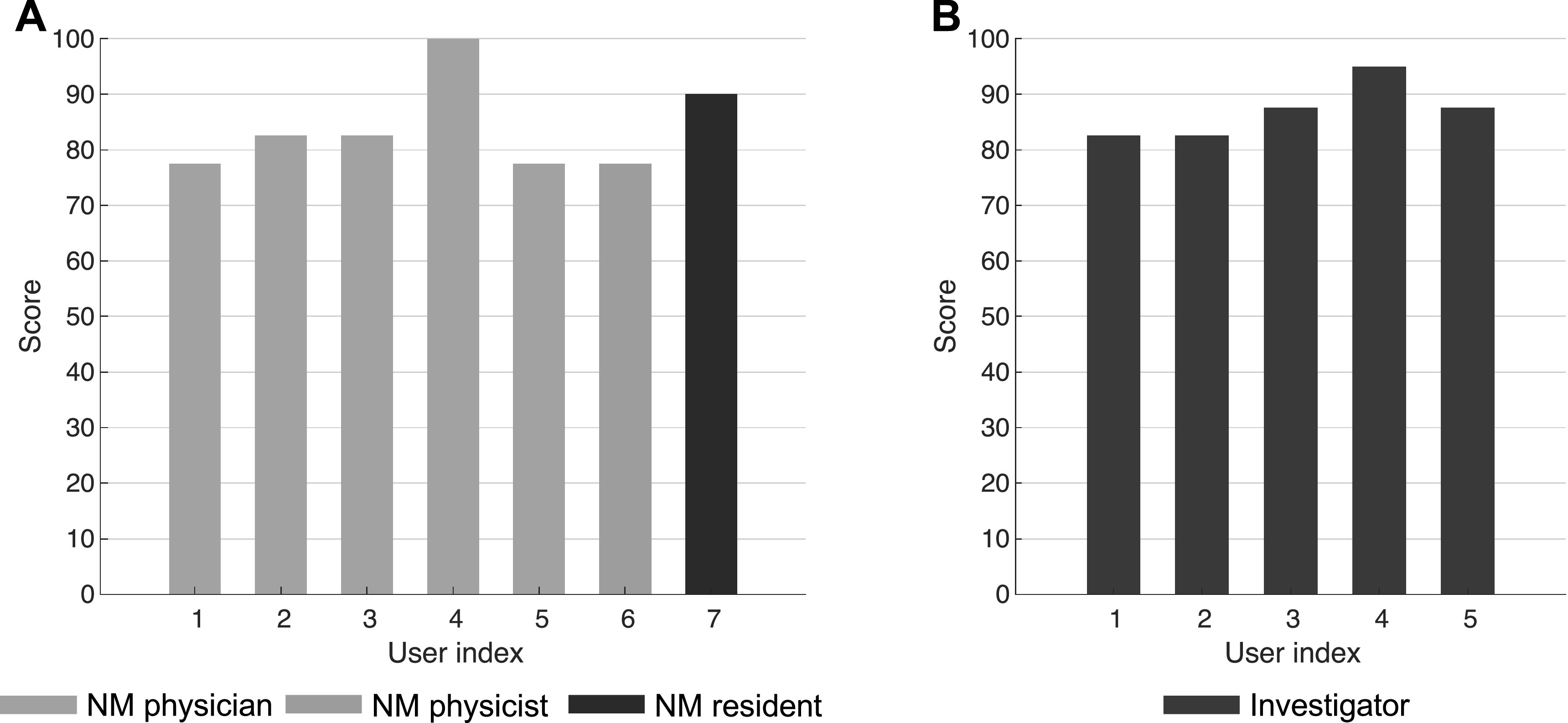
Total score for each user participating in the system usability scale (SUS) survey. (NM: nuclear medicine).

In this section, we report the outcome of the SUS survey conducted to evaluate the usability of the developed web application (section 3.2). Figure 4 presents the distribution of responses from (A) seven expert human readers and (B) five observer-study designers to each item in the questionnaire described in table 1. Figure 5 shows the total score computed for each user based on the rule defined in section 3.2. For the group of expert human readers, a mean score of 84 with standard deviation of 8 was observed. Similarly, a mean score of 87 with standard deviation of 5 was obtained for the group of investigators. Based on Lewis and Sauro (2018), these results indicate that our software is very highly usable.

### 4.2. Evaluating the clinical realism of a PET image-synthesis technique using the developed software

Table 2 shows the percent accuracy and median confidence level for each expert human observer participating in the 2-AFC study to evaluate the clinical realism of the stochastic and physics-based image-synthesis technique using our developed software, as described in section 3.3. We observe that all the readers identified the real PET image correctly only ∼50% of the time. Additionally, for half of the readers, the median value of confidence levels was ≤3.

**Table 2.** Percent accuracy and median confidence level for each expert reader participating in the 2-AFC study.

Reader	Percent accuracy	Median confidence level
PET physician 1	44%	2
PET physician 2	58%	4
PET physician 3	50%	2
PET physician 4	58%	3
PET physician 5	44%	4
PET physicist	58%	4

**Figure 6.**
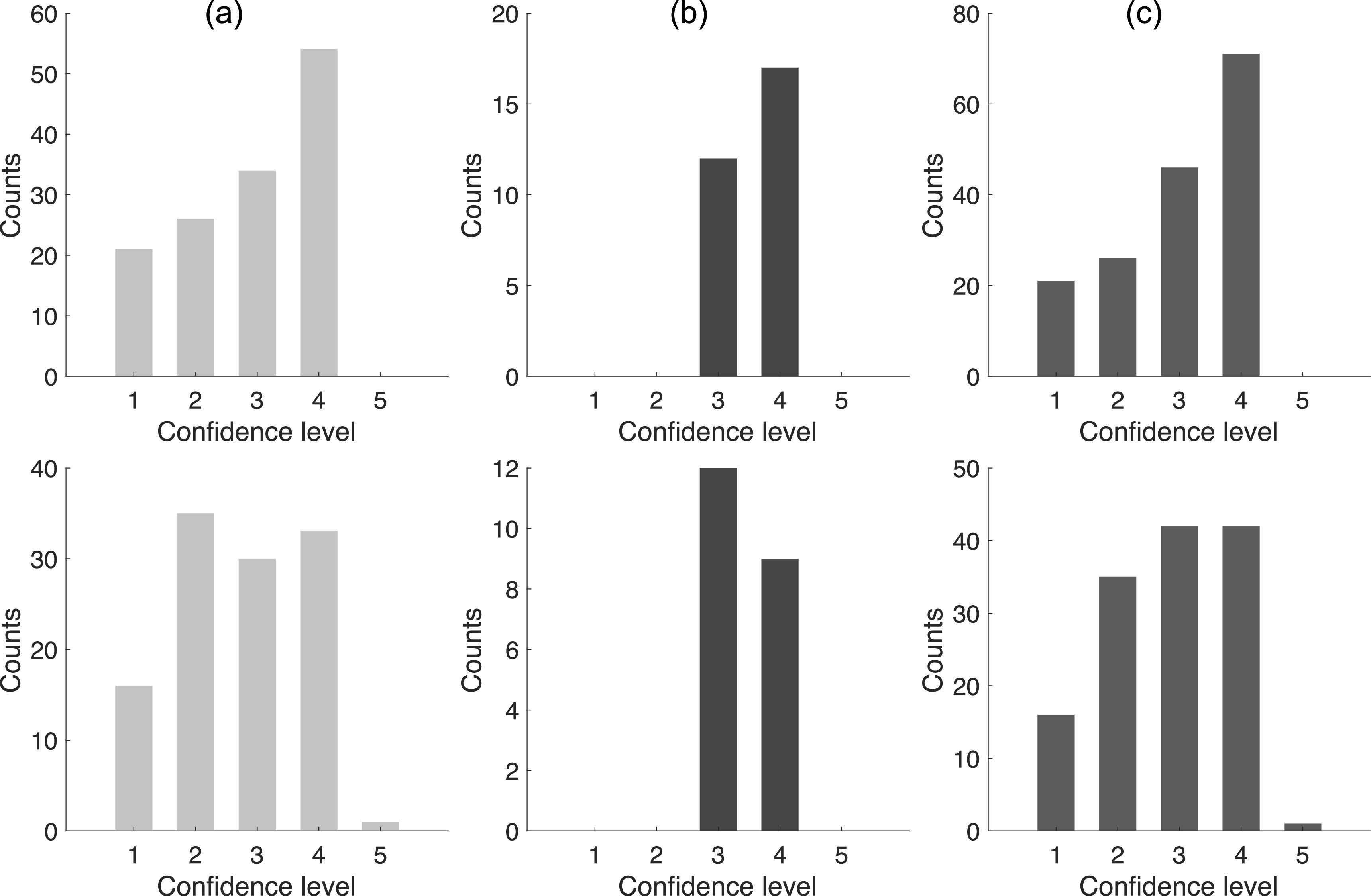
Number of correct (upper row) and incorrect (lower row) decisions made by the (a) five PET physicians, (b) one physicist, and (c) all the readers, at each confidence level.

Figure 6 shows the number of correct (upper row) and incorrect (lower row) decisions made by the (a) five PET physicians, (b) the PET physicist, and (c) all the readers, respectively, at each confidence level. When combining all the readers, only 164/300 (55%) decisions were made correctly. Among these correct decisions, only 71 (43%) were made with confidence levels ≥4. Additionally, 34/136 (25%) incorrect decisions were made with high confidence levels ≥4.

## 5. Discussion

To ensure that simulation-based development and evaluation of medical imaging methods are clinically relevant, images generated by the synthesis technique must be clinically realistic and, ideally, have the same distribution as that of real images. The first contribution of this work is to theoretically demonstrate that an ideal-observer-study-based approach provides a mechanism to quantitatively evaluate the similarity in distributions between the real and synthetic images. Further, we show that the AUC for an ideal observer can be expressed, to an excellent approximation, by the Bhattacharyya distance between the distributions of real and synthetic images. Thus, when the ideal-observer AUC decreases, this indicates that the distance between the two distributions decreases. Moreover, a lower bound of AUC = 0.5 indicates that the distribution of the synthetic images exactly matches that of the real images. Thus, by quantifying the similarity in distributions between the real and synthetic images, this ideal-observer-study-based approach provides a theoretical foundation for quantitative evaluation of the clinical realism of synthetic images.

The second contribution of this manuscript is to develop a web-based platform for facilitating the use of human-observer-study-based approaches to quantitatively evaluate the clinical realism of synthetic images. Our software is openly available, does not require installation on a local workstation, is platform-independent, eliminates the need for on-site study, and allows simultaneous access by multiple users. The goal of incorporating all these features is to strengthen the usability of this software. Additionally, our software provides features that allow varying the contrast and intensity of images. This leads to an user interface that is similar to those present in clinical tools, thus further strengthening the rigor and clinical relevance of the 2-AFC experiments. Our results from the SUS survey shown in section 4.1 demonstrate that the software is highly user-friendly and accessible. Further, our software provides multiple features to align with the General Data Protection Regulation policies. Specifically, the software provides mechanisms to secure stored data, allow users to delete uploaded data, and prevent data from unauthorized access. All these features are important for evaluation studies that include patient data.

Our developed software can be used to evaluate a large class of image-synthesis techniques, including physics-based methods (Duchateau *et al* 2017, Ma *et al* 2017, Leung *et al* 2020, Hamdi *et al* 2021), generative adversarial network-based methods (Costa *et al* 2017, Nie *et al* 2017, Wang *et al* 2021), and other AI-based methods (Chartsias *et al* 2017b, Xiang *et al* 2018, Bahrami *et al* 2020, Dutta *et al* 2022). Further, while the key purpose of our software is evaluating the realism of synthetic images, the software can also be used to conduct 2-AFC experiments for performing image-quality assessment. For this secondary purpose, tools have been developed previously (Vuong *et al* 2018, Genske and Jahnke 2022). Similar to those tools, our software can be used to evaluate newly developed image-reconstruction and image-processing methods on signal-detection tasks.

Another application of the proposed realism-evaluation strategies is in assessing the realism of synthetic images that are generated for virtual clinical trials. For this application, it is important to account for the clinical task of interest and not just assess whether the images look realistic to a human observer (Badano 2017). In that context, our ideal-observer-study-based approach provides a mechanism to quantify the difference in distributions of real and synthetic images. Further, performance on clinical tasks of interest typically depends on the distribution of the image. Future research may reveal that having a measure of the difference between the distributions of real and synthetic images can help to objectively compare the performance on the clinical task with those images. In that case, our theoretical formalism could provide a mechanism to account for the clinical task of interest when evaluating the realism of synthetic images.

As a secondary finding of this work, our evaluation of a stochastic and physics-based image-synthesis technique (section 3.3) using the expert-human-observer-based study with the developed software indicates that the expert readers had limited ability to distinguish the real images from the synthetic images. As shown in table 2, all the expert readers, even including the most experienced PET physician with 40 years of reading PET scans, correctly identified the real images only in ∼50% of the cases. Additionally, we observe from figure 6 that among the 164 (out of 300) correct decisions, only 43% were made with high confidence levels, suggesting that the readers were not confident even when they correctly identified the real image. Moreover, the readers were falsely confident for 25% of incorrect decisions. These results motivate the use of the image-synthesis technique to generate images for the development and evaluation of a wide range of PET imaging methods. In fact, this technique was used to objectively evaluate a recently developed PET segmentation method (Liu *et al* 2021b).

There are some limitations in this work. First, our ideal-observer-study-based approach to evaluate the clinical realism of synthetic images was presented in theory and not yet applied to a clinical scenario. As shown in section 2, developing the ideal observer requires knowledge of the probability distributions of the real and synthetic images. However, in clinical studies, these distributions are high-dimensional and do not have a known analytical form. To address these issues, AI-based methods are showing promise in approximating the ideal-observer test statistics for signal-detection tasks (Kupinski *et al* 2001, Zhou *et al* 2019b). Our theoretical formalism motivates extending these methods for the task of clinical realism evaluation. Second, our theoretical formalism was presented specifically for an ideal observer and thus, we reiterate that it should not be used to directly interpret results obtained with expert human observers. However, in that context, we do point out that several studies (He *et al* 2004, Li *et al* 2016) have shown correlations between the performance of human observers and channelized Hotelling observers (CHOs). The CHOs utilize templates that are derived from the first- and second-order statistics of the channel vectors extracted from the images. Thus, in special cases where the channel vectors are sufficient statistics for describing the distributions of real and synthetic images, our ideal-observer analysis may be used to quantify the similarity in distributions of real and synthetic images. Examining this connection is an important future research direction. A third limitation is that our web application is currently designed to evaluate the realism of synthetic images on a per-slice basis and not the entire 3D volume. Additionally, in the designed application, the slices are displayed only in a single orientation. Expanding the web application to display images in 3D and in multiple orientations is an important area of future development. Finally, our web application is currently developed for conducting 2-AFC experiments. Considering that different variants of the 2-AFC experiment have been used in the human-observer studies (Zhang *et al* 2016, Ikejimba *et al* 2019), expanding our software to allow conducting those experiments is another important area of future development.

## 6. Conclusion

In this work, we investigated two observer-study-based approaches to quantitatively evaluate the clinical realism of synthetic images. We theoretically demonstrated that an ideal-observer-study-based approach provides a mechanism to quantify the similarity in distributions of real and synthetic images. Further, we showed that the ideal-observer AUC can be expressed, to an excellent approximation, by the Bhattacharyya distance between the distributions of real and synthetic images. Additionally, we developed a software that provides a web-based platform to facilitate the conducting of expert-human-observer studies for quantitative evaluation of the realism of synthetic images. This software is available at https://apps.mir.wustl.edu/twoafc. The software provides multiple functionalities towards increasing the rigor and clinical relevance of 2-AFC experiments. Our results from the SUS survey demonstrate that this software enables designing and performing 2-AFC experiments with expert human observers in a highly accessible and user-friendly manner. Finally, as a secondary finding of this work, evaluation of a stochastic and physics-based PET image-synthesis technique showed that the expert human observers were generally unable to distinguish the real images from the synthetic images. This finding motivates the application of this technique to the development and evaluation of PET imaging methods.

## Ethical statement

The study was conducted under an approved IRB protocol titled ‘PET Radiomics of NSCLC’ (ID: 201906021) at Washington University in St. Louis. We confirm that the research was conducted in accordance with the principles embodied in the Declaration of Helsinki and in accordance with local statutory requirements. This was a retrospective study and waiver of consent was granted.

## Appendix A

In this appendix, we prove that when an observer performs a 2-AFC experiment, the expression for the probability of a correct decision (equation (1)) is equal to the AUC for that observer. Our proof is similar to that provided in Barrett *et al* (1998) but for a different context. In that paper, the derivation was presented in the context of performing a 2-AFC study to evaluate the observer performance for a signal-detection task. Here, we paraphrase the derivation for the application of evaluating the clinical realism of synthetic images.

Proof. Consider an observer performing the task of identifying an image as synthetic (*H*_1_) or real (*H*_2_). For a given image, the observer calculates a test statistic, denoted by a random variable *t*, and then compares the value of *t* to a threshold, denoted by *x*. If $t\geqslant x$, the observer will identify the image as real, i.e. assign the image to *H*_2_. Otherwise, the image is considered synthetic and assigned to *H*_1_. The performance of this observer can be fully specified by two quantities. The first quantity, referred to as the true-positive fraction (TPF), measures the fraction of times that the observer identifies the image as real when the image is indeed real. The second quantity, referred to as false-positive fraction (FPF), measures the fraction of times that the observer identifies the image as real when the image is in fact synthetic. Denote the probability of an event by $\Pr (\cdot )$ and the probability distribution of a random variable by $\mathrm{pr}(\cdot )$. Given the threshold *x*, the TPF and FPF can be calculated as follows:

\begin{eqnarray*}\mathrm{TPF}(x)=\Pr (t\geqslant x| {H}_{2})={\int }_{x}^{\infty }{\mathrm{d}}{t}\ \mathrm{pr}(t| {H}_{2}),\end{eqnarray*}

\begin{eqnarray*}\mathrm{FPF}(x)=\Pr (t\geqslant x| {H}_{1})={\int }_{x}^{\infty }{\mathrm{d}}{t}\ \mathrm{pr}(t| {H}_{1}).\end{eqnarray*}

Then, the expression for the $\mathrm{AUC}$ can be obtained in terms of the $\mathrm{TPF}$ and $\mathrm{FPF}$ as

\begin{eqnarray*}\mathrm{AUC}={\int }_{0}^{1}{\mathrm{d}}\mathrm{FPF}(x)\ \mathrm{TPF}(x)\end{eqnarray*}

\begin{eqnarray*}=\,\,-{\int }_{-\infty }^{\infty }{\mathrm{d}}{x}\ \displaystyle \frac{{\mathrm{d}}}{{\mathrm{d}}{x}}\mathrm{FPF}(x)\mathrm{TPF}(x),\end{eqnarray*}

where, in the second step, we have changed the variable of integration from $\mathrm{FPF}(x)$ to *x*. For convenience of notation, we define ${p}_{j}(t)\equiv \mathrm{pr}(t| {H}_{j})$. Using equation (A.1b) and Leibniz’s rule, we have

\begin{eqnarray*}\displaystyle \frac{{\mathrm{d}}}{{\mathrm{d}}{x}}\mathrm{FPF}(x)=-{p}_{1}(x).\end{eqnarray*}

Then, we can re-write equation (A.2b) as

\begin{eqnarray*}\mathrm{AUC}={\int }_{-\infty }^{\infty }{\mathrm{d}}{x}\ {p}_{1}(x){\int }_{x}^{\infty }{\mathrm{d}}{t}\ {p}_{2}(t)\end{eqnarray*}

\begin{eqnarray*}=\,{\int }_{-\infty }^{\infty }{\mathrm{d}}{x}\ {\int }_{-\infty }^{\infty }{\mathrm{d}}{t}\ {p}_{1}(x){p}_{2}(t)\mathrm{step}(t-x).\end{eqnarray*}

Since the test statistic is calculated based on the image $\hat{{\bf{f}}}$, we consider *t* as a function of $\hat{{\bf{f}}}$ such that $t=\theta (\hat{{\bf{f}}})$. Thus, we can further write the AUC in equation (A.4) in terms of integrals over $\hat{{\bf{f}}}$. For this purpose, we first express the term *p*_*j*_(*t*) as

\begin{eqnarray*}{p}_{j}(t)={\int }_{\infty }{{\mathrm{d}}}^{M}\hat{{\bf{f}}}\ \mathrm{pr}(t| \hat{{\bf{f}}})\mathrm{pr}(\hat{{\bf{f}}}| {H}_{j})\end{eqnarray*}

\begin{eqnarray*}=\,{\int }_{\infty }{{\mathrm{d}}}^{M}\hat{{\bf{f}}}\ \mathrm{pr}(\hat{{\bf{f}}}| {H}_{j})\delta [t-\theta (\hat{{\bf{f}}})],\end{eqnarray*}

where, in the second step, the function $t=\theta (\hat{{\bf{f}}})$ is represented as a probabilistic mapping. For convenience of notation, we define ${q}_{j}(\hat{{\bf{f}}})\equiv \mathrm{pr}(\hat{{\bf{f}}}| {H}_{j})$. Then, from equations (A.4b) and (A.5), we obtain

\begin{eqnarray*}\mathrm{AUC}={\int }_{-\infty }^{\infty }{\mathrm{d}}{x}\ {\int }_{-\infty }^{\infty }{\mathrm{d}}{t}\ {\int }_{\infty }{{\mathrm{d}}}^{M}\hat{{\bf{f}}}\ {q}_{1}(\hat{{\bf{f}}})\delta [x-\theta (\hat{{\bf{f}}})]{\int }_{\infty }{{\mathrm{d}}}^{M}\hat{{\bf{f}}}^{\prime} \ {q}_{2}(\hat{{\bf{f}}}^{\prime} )\delta [t-\theta (\hat{{\bf{f}}}^{\prime} )]\mathrm{step}(t-x)\end{eqnarray*}

\begin{eqnarray*}=\,{\int }_{\infty }{{\mathrm{d}}}^{M}\hat{{\bf{f}}}\ {q}_{1}(\hat{{\bf{f}}}){\int }_{\infty }{{\mathrm{d}}}^{M}\hat{{\bf{f}}}^{\prime} \ {q}_{2}(\hat{{\bf{f}}}^{\prime} ){\int }_{-\infty }^{\infty }{\mathrm{d}}{x}\ \delta [x-\theta (\hat{{\bf{f}}})]{\int }_{-\infty }^{\infty }{\mathrm{d}}{t}\ \delta [t-\theta (\hat{{\bf{f}}}^{\prime} )]\mathrm{step}(t-x)\end{eqnarray*}

\begin{eqnarray*}=\,{\int }_{\infty }{{\mathrm{d}}}^{M}\hat{{\bf{f}}}{\int }_{\infty }{{\mathrm{d}}}^{M}\hat{{\bf{f}}}^{\prime} \ {q}_{1}(\hat{{\bf{f}}}){q}_{2}(\hat{{\bf{f}}}^{\prime} )\mathrm{step}(\theta (\hat{{\bf{f}}}^{\prime} )-\theta (\hat{{\bf{f}}})),\end{eqnarray*}

where in the second step, we have interchanged the order of integration and, in the third step, we have used the sifting property of the delta function to perform the integrals over *x* and *t*. We then immediately see that equation (A.6c) has the same form as in equation (1).□

## Appendix B

In this appendix, we provide the derivation for obtaining the relationship between the probability distribution of the log-likelihood ratio *λ* under the two hypothesis (equation (10)).

We first apply inverse Fourier transform to equation (9) on both sides:

\begin{eqnarray*}{p}_{2}(\lambda )={\int }_{-\infty }^{\infty }{\mathrm{d}}\xi \ {\psi }_{1}\left(\xi +\displaystyle \frac{{\mathrm{i}}}{2\pi }\right)\exp (2\pi {\mathrm{i}}\xi \lambda ).\end{eqnarray*}

By letting $z=\xi +\tfrac{{\mathrm{i}}}{2\pi }$, we have

\begin{eqnarray*}{p}_{2}(\lambda )=\exp (\lambda ){\int }_{-\infty +\tfrac{{\mathrm{i}}}{2\pi }}^{\infty +\tfrac{{\mathrm{i}}}{2\pi }}{\mathrm{d}}{z}\ {\psi }_{1}(z)\exp (2\pi {\mathrm{i}}{z}\lambda ).\end{eqnarray*}

As proven in Barrett *et al* (1998), the contour in equation (B.2) can be shifted as long as 〈Λ〉_2_ is finite, thus, yielding

\begin{eqnarray*}{p}_{2}(\lambda )=\exp (\lambda ){\int }_{-\infty }^{\infty }{\mathrm{d}}{z}\ {\psi }_{1}(z)\exp (2\pi {\mathrm{i}}{z}\lambda )\end{eqnarray*}

\begin{eqnarray*}=\,\exp (\lambda ){p}_{1}(\lambda ).\end{eqnarray*}

## Appendix C

In this appendix, we describe the programming environment of the developed web application.

The web application was developed on Microsoft's .NET 6 software framework and leveraged the Razor Pages web development model. The Razor Pages model incorporates the model-view-viewmodel design pattern to facilitate separation of the user-interface layer from the backend domain layer. The model-view-viewmodel pattern is an object-oriented-programming paradigm characterized by the use of an intermediary viewmodel object that serves to expose data within model objects for presentation to the user within the view. The application is primarily written in the C# programming language for server-side operations, and adopts the object-oriented-programming approach. The application also consists of a client-side layer written in JavaScript to deliver responsive and dynamic user-interface functionalities to the user. The client-side layer integrates data retrieved from the server with the user-interface to create functionality that is not dependent on additional HTTP requests to render. The Module Pattern is used to scope client-side code to defined areas within the application and encapsulate application logic. Application data is persisted within a SQL Server relational database instance which communicates with the application through Microsoft's Entity Framework object-relational-mapping tool. A Microsoft Azure B2C tenant instance was employed to handle user access and authentication into the application. The B2C tenant also handles third-party authentication for the application.
